# Clinical Outcomes of Patients with Resected Oral Cavity Cancer and Simultaneous Second Primary Malignancies

**DOI:** 10.1371/journal.pone.0136918

**Published:** 2015-09-03

**Authors:** Chun-Ta Liao, Kang-Hsing Fan, Chung-Jan Kang, Chien-Yu Lin, Joseph Tung-Chieh Chang, Ngan-Ming Tsang, Bing-Shen Huang, Yin-Kai Chao, Li-Yu Lee, Chuen Hsueh, Hung-Ming Wang, Chi-Ting Liau, Cheng-Lung Hsu, Chia-Hsun Hsieh, Shu-Hang Ng, Chih-Hung Lin, Chung-Kan Tsao, Tuan-Jen Fang, Shiang-Fu Huang, Kai-Ping Chang, Tzu-Chen Yen

**Affiliations:** 1 Department of Otorhinolaryngology, Head and Neck Surgery, Chang Gung Memorial Hospital and Chang Gung University, Taoyuan, Taiwan, ROC; 2 Head and Neck Oncology Group, Chang Gung Memorial Hospital and Chang Gung University, Taoyuan, Taiwan, ROC; 3 Department of Radiation Oncology, Chang Gung Memorial Hospital and Chang Gung University, Taoyuan, Taiwan, ROC; 4 Department of Thoracic and Cardiovascular Surgery, Chang Gung Memorial Hospital and Chang Gung University, Taoyuan, Taiwan, ROC; 5 Department of Pathology, Chang Gung Memorial Hospital and Chang Gung University, Taoyuan, Taiwan, ROC; 6 Department of Medical Oncology, Chang Gung Memorial Hospital and Chang Gung University, Taoyuan, Taiwan, ROC; 7 Department of Diagnostic Radiology, Chang Gung Memorial Hospital and Chang Gung University, Taoyuan, Taiwan, ROC; 8 Department of Plastic and Reconstructive Surgery, Chang Gung Memorial Hospital and Chang Gung University, Taoyuan, Taiwan, ROC; 9 Department of Nuclear Medicine and Molecular Imaging Center, Chang Gung Memorial Hospital and Chang Gung University, Taoyuan, Taiwan, ROC; University of North Carolina School of Medicine, UNITED STATES

## Abstract

**Objectives:**

Simultaneous second primary tumors (SSPT) are not uncommon in patients with oral cavity squamous cell carcinoma (OSCC) living in areas where the habit of betel quid chewing is widespread. We sought to identify the main prognostic factors in OSCC patients with SSPT and incorporate them into a risk stratification scheme.

**Methods:**

A total of 1822 consecutive patients with primary OSCC treated between January 1996 and February 2014 were analyzed for the presence of SSPT. The 18-month and 5-year overall survival (OS) rates served as the main outcome measures.

**Results:**

Of the 1822 patients, 77 (4%) were found to have SSPT (i.e, two malignancies identified within one month of each other). The 18-month and 5-year OS rates in patients without SSPT and with SSPT were 82% and 69%, and 72% and 53%, respectively (*p* = 0.0063). Patients with SSPT were further divided into patients with either esophageal cancer or hepatocellular carcinoma (eso-HCC subgroup, n = 8) and other tumors (NO eso-HCC subgroup, n = 69). After multivariate analysis, neck nodal extracapsular spread (ECS, n = 18) and the presence of eso-HCC were identified as independent adverse prognostic factors. The 18-month OS rates of SSPT patients with both eso-HCC and ECS (n = 5) *vs*. the remaining patients (n = 72) were 0% and 78%, respectively (*p* < 0.0001).

**Conclusion:**

OSCC patients with neck nodal ECS and esophageal cancer or hepatocellular carcinoma as SSPT have a dismal short-term prognosis.

## Introduction

Simultaneous second primary tumors (SSPT) are not uncommon in patients with oral cavity squamous cell carcinoma (OSCC) [1], especially in areas where the habit of betel quid chewing is widespread [2]. We and others have previously shown that OSCC patients with SSPT generally have a poor prognosis [2–4]. However, the clinical outcomes of patients with first primary OSCC may be dependent on the presence of neck nodal extracapsular spread (ECS, a major adverse prognostic factor in OSCC) [5] and/or the site of second primary tumors (SPT; e.g., esophagus, hypopharynx, or lung) [6]. Radical surgery with or without postoperative adjuvant therapy (depending on the presence of pathological risk factors) remains the mainstay of treatment for OSCC patients. A secondary treatment strategy should be planned in OSCC patients who present with SSPT at the time of primary treatment [7,8].

According to the Taiwanese 2011 official statistics, liver, lung, hypopharyngeal, and esophageal malignancies rank first, second, fourth, and fifth, respectively, as the leading causes of cancer-related death in the male population [9]. Moreover, Taiwan is characterized by a markedly high incidence of HBV- and HCV-related hepatocellular carcinoma (HCC). Of note, approximately 5% of our OSCC patients present with concomitant HCC. However, the question as to whether OSCC patients with SSPT located at the liver or other at-risk sites should receive specific and/or targeted treatment approaches remains open [7,8]. In this scenario, we designed the current study to identify the main prognostic factors in OSCC patients with SSPT and incorporate them into a risk stratification scheme.

## Patients and Methods

### Patients

Between January 1996 and February 2014, we identified a total of 1822 consecutive untreated patients presenting with first primary OSCC who were scheduled for radical surgery, either with or without neck dissection (ND). All of the participants underwent an extensive presurgical evaluation and staging workup. As of October 2002, the majority of the study patients underwent preoperative panendoscopy. Starting from August 2001, most patients with stage II-IV disease received whole-body FDG-PET for primary staging. Patients were staged according to the 1997 (5^th^) and 2010 (7^th^) staging criteria of the American Joint Committee on Cancer (AJCC). The 1997 criteria were used for patients enrolled before 2002, whereas the 2010 criteria were utilized for patients recruited after 2002. The major difference between the two staging systems is that some tumors with invasion of the masticator space/pterygoid plate would be classified as pT4b using the AJCC 2010 criteria, but only as pT2-T3 according to the 1997 criteria [10]. If two separated oral cavity malignancies were detected simultaneously, the more advanced-staged tumor was considered as the index malignancy. The study protocol was approved by the Institutional Review Board of the Chang Gung Memorial Hospital (CGMH 101-4457B). Patient consent was waived due to the retrospective nature of the study.

### Surgery and adjuvant therapy

The primary tumors were excised with safety margins of 1 cm or greater (both peripheral and deep margins). Level I–V NDs were performed in patients with cN+ disease, whereas cN- patients received level I–III NDs [2,5,10]. In general, post-operative radiotherapy (RT, 60 Gy) was performed for patients bearing pathological risk factors (RFs). RFs were classified according to the NCCN guidelines before 2008; thereafter, RFs classification was based on the Chang Gung guidelines outlined in our previous publications [11]. The main RFs for RT included: pT4, pT3N1, pT1-2N1 (N1 at levels IV/V), close margins ≤2 mm, poor differentiation with tumor depth ≥4 mm. Otherwise, the presence of at least 2 minor RFs (i.e., pN1, tumor depth ≥10 mm, close margins ≤4 mm, poor differentiation, perineural invasion, lymphatic invasion, vascular invasion) were required for RT. The radiation field included the entire tumor bed area (with 1- to 2-cm margins) as well as the regional lymphatics. Concomitant chemoradiation (CCRT, 66 Gy) with cisplatin-based regimens was administered to patients with ECS, multiple lymph node metastases, positive margins, or bearing at least three minor risk factors (i.e., the above-mentioned minor RFs plus pT4) [12–14]. The chemotherapy regimen consisted of intravenous cisplatin 50 mg/m^2^ biweekly plus daily oral tegafur 800 mg and leucovorin 60 mg, cisplatin 40 mg/m^2^ weekly, or cisplatin 100 mg/m^2^ every 3 weeks [14].

### Definitions and data analysis

SPT were defined as malignancies that were both distinct and anatomically separated (i.e., having at least 2 cm of normal tissue between each lesion). Metastases or local relapses were carefully excluded. Similarly, tumors occurring at the same site (regardless of the time elapsed from the patient’s first definitive treatment) were not considered as SPT. SSPT were defined as documented malignancies occurring within one month from OSCC diagnosis, whereas not-SSPT were considered to be present when tumors were identified after at least one month from the initial OSCC diagnosis. Follow-up was continued until February 2015. All of the study patients received follow-up examinations for at least 12 months after primary definitive treatment for OSCC or until death. The 18-month and 5-year overall survival (OS) rates served as the main outcome measure. OS was calculated from the date of surgery to the date of death or the last follow-up. Survival curves were plotted using the Kaplan—Meier method and compared with the log-rank test. Univariate and multivariate analyses (UVA and MVA) were used to identify the main prognostic factors. MVA was based on the Cox logistic regression method with a forward selection procedure. All calculations were performed using the SPSS 17.0 statistical software (SPSS Inc., Chicago, IL, USA). Two-tailed *p* values <0.05 were considered statistically significant. All relevant data are within the paper and its supporting information S1 and S2 Data.

## Results

### Patient characteristics and clinical outcomes

**Fig 1:**
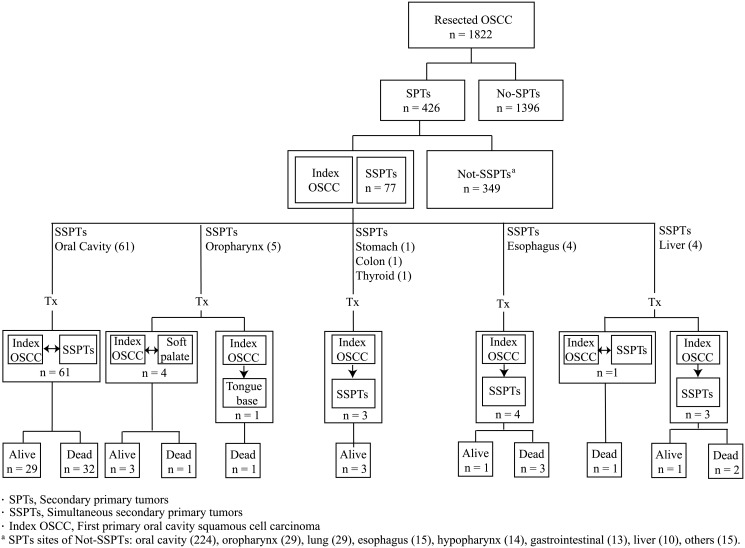
Clinical and demographic characteristics of the study patients summarizing the treatment modalities and the clinical outcomes of OSCC patients presenting with SSPT.

**Fig 2:**
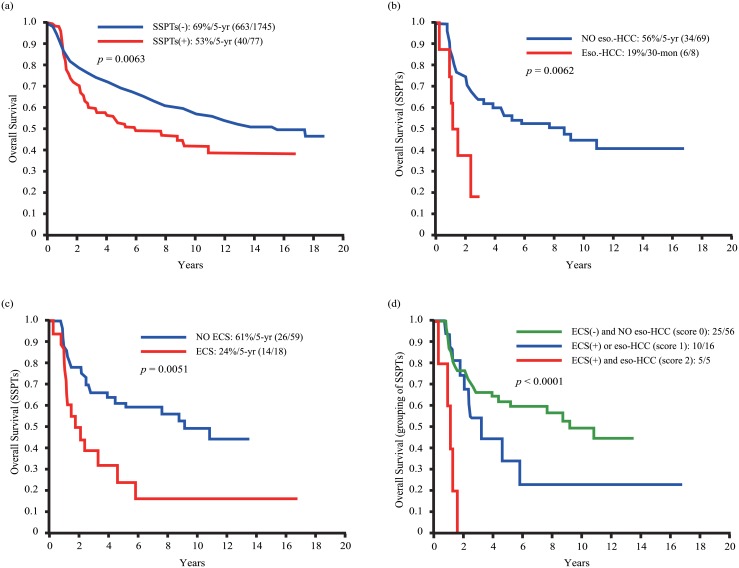
Kaplan-Meier plots of 5-year rates of overall survival in OSCC patients with and without SSPT (a); patients stratified according to the presence of SSPT located at the esophagus or the liver (b), extracapsular spread (c), and both risk factors (d).

Table 1 depicts the general characteristics of the study participants. Of the 1822 patients, 426 (23%) were found to have SPT (77 [4%] SSPT, 349 (19%) not-SSPT), and 1396 (77%) no-SPT (Fig 1, upper panel). The rate of SPT within the first month of diagnosis (i.e., SSPT) of the index OSCC was 4%, with an annual increase of approximately 3% (20%/5-year, 34%/10-year). The 18-month and 5-year OS rates in the entire cohort were 81% and 68%, respectively. The 18-month and 5-year OS rates in patients without SSPT (i.e., no-SPT plus not-SSPT, n = 1745) and with SSPT were 82% and 69%, and 72% and 53%, respectively (*p* = 0.0063, Fig 2a). Table 2 (left part) depicts the general characteristics of OSCC patients with SSPT. All of the 77 patients with SSPTs were male. The age at onset ranged between 29 and 73 years (mean: 53 years, median: 53 years). The distribution of risky oral habits was as follows: 61 patients (79%) had a history of preoperative alcohol drinking, 69 (90%) of preoperative betel chewing, and 69 (90%) of preoperative cigarette smoking. The sites of SSPT were as follows: oral cavity (n = 61, 79%), oral pharynx (n = 5, 7%), esophagus (n = 4, 5%), liver (HCC, n = 4, 5%), stomach (n = 1, 1%), colon (n = 1, 1%), and thyroid (n = 1, 1%). Regarding the treatment modality for the index OSCC, 31 patients (40%) had surgery alone, 29 (38%) received surgery plus RT, and 17 (22%) received surgery plus CCRT.

**Table 1:** Clinicopathological characteristics of OSCC patients undergoing surgery (n = 1822).

Characteristics	Number of patients (%)	
	n	%
Sex		
Male	1701	93.4
Female	121	6.6
Age at onset (years)		
Range: 25–89 (median 51)		
< 65	1578	86.6
≥ 65	244	13.4
Pathological T-status		
pT1	339	18.6
pT2	754	41.4
pT3	303	16.6
pT4	426	23.4
Pathological N-status		
pNx (no neck dissection)	125	6.9
pN0	1044	57.3
pN1	228	12.5
pN2	425	23.3
Pathological stage^a^		
I	303	16.6
II	495	27.2
III	333	18.3
IV	691	37.9
Extracapsular spread^b^		
No	1437	79.0
Yes	382	21.0
Tumor differentiation		
Well	698	38.3
Moderate	964	52.9
Poor	160	8.8
Tumor depth (mm)^b^		
< 10	941	51.8
≥ 10	877	48.2
Margin status (mm)^b^		
≤ 4	190	10.5
> 4	1616	89.5
Bone marrow invasion		
No	1569	86.1
Yes	253	13.9
Skin invasion		
No	1689	92.7
Yes	133	7.3
Perineural invasion^b^		
No	1261	69.2
Yes	560	30.8
Lymphatic invasion^b^		
No	1726	94.8
Yes	94	5.2
Vascular invasion^b^		
No	1776	97.6
Yes	44	2.4
Treatment modality		
Surgery alone	886	48.6
Surgery plus RT	506	27.8
Surgery plus CCRT	430	23.6

Abbreviations: RT, radiotherapy; CCRT, concurrent chemoradiotherapy.

^a^Patients who did not undergo neck dissection were classified as pN0.

^b^Unavailable data: extracapsular spread (n = 3), tumor depth (n = 4), margin status (n = 16), perineural invasion (n = 1), lymphatic invasion (n = 2), vascular invasion (n = 2).

**Table 2:** Univariate and multivariate analyses of 5-year overall survival in OSCC patients with SSPT (n = 77).

Characteristics	Number of patients (%)		5-year overall survival			
	n	%	Univariate			Multivariate
			5-year %	Number of events	*p*	*p*, HR (95% CI)
Esophagus or liver subsites					0.0062	0.030, 2.829 (1.108–7.220)
No	69	89.6	56	34		
Yes	8	10.4	19	6		
Sex					-	-
Male	77	100.0	53	40		
Age at onset (years)					0.9162	ns
< 65	64	83.1	52	33		
≥ 65	13	16.9	53	7		
Pathological T-status					0.2838	ns
pT1	2	2.6	50	1		
pT2	31	40.3	63	13		
pT3	21	27.3	52	10		
pT4	23	29.9	40	16		
Pathological N-status					0.0113	ns
pN0	49	66.2	64	21		
pN1	7	9.5	38	4		
pN2	18	24.3	24	14		
Pathological stage^a^					0.1971	ns
I	2	2.6	50	1		
II	21	27.3	71	7		
III	22	28.6	50	11		
IV	32	41.6	44	21		
Extracapsular spread					0.0051	0.018, 2.273(1.153–4.483)
No	59	76.6	61	26		
Yes	18	23.4	24	14		
Tumor differentiation					0.3190	ns
Well	29	37.7	61	13		
Moderate	43	55.8	47	25		
Poor	5	6.5	60	2		
Tumor depth (mm)					0.4392	ns
< 10	32	41.6	61	15		
≥ 10	45	58.4	46	25		
Margin status (mm)^b^					0.0717	ns
≤ 4	10	13.2	30	8		
> 4	66	86.8	57	31		
Bone marrow invasion					0.1038	ns
No	57	74.0	56	26		
Yes	20	26.0	42	14		
Skin invasion					0.3956	ns
No	69	89.6	55	35		
Yes	8	10.4	38	5		
Perineural invasion					0.2312	ns
No	54	70.1	54	26		
Yes	23	29.9	50	14		
Lymphatic invasion					0.0168	ns
No	71	92.2	56	35		
Yes	6	7.8	17	5		
Vascular invasion					-	-
No	77	100.0	53	40		
Third primary tumor					0.1960	ns
Without	48	62.3	47	26		
With	29	37.7	62	14		

Abbreviations: SSPT, simultaneous second primary tumors; HR, hazard ratio; CI, confidence interval; ns, not significant.

^a^Patients who did not undergo neck dissection (n = 3) were classified as pN0.

^b^Unavailable data: margin status (n = 1)

All of the 77 OSCC patients with SSPT were followed up for at least 12 months after primary surgery or until death (mean: 58 months, median: 34 months, range: 3–202 months). At the end of the study period, 37 patients (48%) were alive and 40 (52%) were dead. The patterns of recurrence for the index OSCC and rate of third primary tumors were as follows: local recurrence, 12% (n = 9); neck recurrence, 9% (n = 7); distant metastases, 10% (n = 8) and third primary tumors, 38% (n = 29). Salvage therapy for the primary OSCC was performed in six (40%) of the 15 patients with local and/or neck recurrences (one patient had both local and neck recurrence). Among the patients who were salvaged, two (33.3%) were still alive when the data were analyzed, whereas the remaining four (66.7%) were dead.

Fig 1 depicts the flow of the patients through the study and their clinical outcomes. All of the 77 OSCC patients with SSPT received radical primary tumor excision accompanied either by simultaneous removal of SSPT (n = 66; oral cavity [n = 61], oropharynx [soft palate, n = 4], liver [n = 1]) or subsequent treatment of SSPT at follow-up (n = 11; oropharynx [tongue base, n = 1], stomach [n = 1], colon [n = 1], thyroid [n = 1], esophagus [n = 4], liver [n = 3]).

Of the 61 patients who received simultaneous radical excision of both the index OSCC tumor and the SSPT in the oral cavity, 29 (47%) patients were alive at the time of analysis, whereas the remaining 32 (53%) were dead. Of the five patients with SSPT located in the oropharynx, four had their SSPT located in the soft palate removed alongside with the index OSCC tumor. In this subgroup of patients, three (75%) subjects were still alive, whereas the remaining one (25%) died. A patient with a SSPT arising at the tongue base received RT after radical excision of the primary OSCC. Unfortunately, the patient died 11 months after surgery because of distant relapse. All of the three patients with SSPT located in the stomach, colon, and thyroid received curative surgery followed by complete treatment of the index OSCC. Their survival following radical surgery was 90, 88, and 30 months, respectively. All of the four patients with SSPT located in the esophagus received sequential treatment. Two patients were treated with RT for both OSCC and SSPT in the esophagus (Table 3, cases 3 and 4). Treatment volume of RT included the tumor bed of OSCC, SSPT in esophagus, and regional lymphatics of OSCC and SSPT in the esophagus (neck, mediastinal, and upper abdominal lymphatics). Radiotherapy was performed according to two different treatment plans. Such plans were given sequentially and junction was carefully matched to avoid overlaps in radiation field and the occurrence of severe complications. One patient (25%) was still alive at the time of analysis, whereas the remaining three (75%) were dead. No recurrences or severe complications were observed at the junction between the two RT plans. Of the four patients with SSPT located in the liver, three received sequential treatment. Of them, one (33%) was still alive at the time of analysis, whereas the remaining two (67%) were dead. One patient underwent simultaneous radical excision of both the index OSCC and the simultaneous esophageal malignancy. This patient died of hepatic failure and gastrointestinal bleeding during RT.

**Table 3:** Clinicopathological characteristics of oral cavity cancer patients presenting with SSPT located at the esophagus or the liver (n = 8).

No	Age, years	Primary treatment	Site	Stage	ECS	Interval between primary surgery and clinical events					
						Tumor recurrence	Neck recurrence	DM	Tumor salvage	DOD	AND
1	50	S to tongue, + CCRT (6600 cGy) to eso.	Tongue	pT2N0	-	-	-	-		-^a^	
			Middle eso.	T1N0						-^a^	
2	60	S to tongue, + S to eso., + RT (6000 cGy) to tongue	Tongue	pT2N1	-	-	-	-			18
			Lower eso.	pT1bN0							18
3	44	S+CCRT (6600 cGy) to bucca, + RT (3000+3000 cGy) to eso.	Buccal	pT2N2b	+	-	-	-		-	
			Middle eso.	T2N0				12	CCRT	18^b^	
4	70	S to mouth floor, + CCRT (6600 cGy) to mouth, floor and eso.	Mouth floor	pT4N2c	+	4	-	-	-	13	
			Upper eso.	T3N1M0						13	
5	69	S+RT (6000 cGy) to tongue, + TACE to liver	Buccal	pT2N0	-	-	-	-			36
			Liver	T2N0							36
6	54	S+CCRT (4000 cGy)^c^ to buccal, No treatment for liver	Buccal	pT4N2b	+	9	-	-	-	14	
			Liver	grade II/III						14	
7	49	S+CCRT (6600 cGy) to tongue, + TACE to liver	Tongue	pT2N2b	+	-	-	-		-	
			Liver	T2N1						11^d^	
8	55	S to retromolar and liver, + RT (3000cGy)^e^ to retromolar	Retromolar	pT4N2b	+	-	-	-		-	
			Liver	grade III						3^5^	

^a^(case 1) Died of third primary squamous cell carcinoma of the tongue base 29 months after primary surgery for OSCC.

^b^(case 3) Died of malignant pleural effusion related to the second primary tumor.

^c^(case 6) Incomplete CCRT due to chemotherapy and liver cirrhosis-induced pancytopenia.

^d^(case 7) Died of upper gastrointestinal bleeding.

^e^(case 8) Incomplete RT due to jaundice and ascites, died of hepatic failure and gastrointestinal bleeding.

Abbreviations: SSPT, simultaneous secondary primary tumor; eso., esophagus; TACE, transcatheter arterial chemoembolization; DM, distant metastases; ECS, extracapsular spread; S, surgery; RT, radiotherapy; CCRT, concurrent chemoradiotherapy; DOD, died of disease or disease-related causes; AND, alive without disease.

### Independent prognostic factors for 5-year OS in OSCC patients with SSPT (n = 77)

The 5-year disease-free survival and disease-specific survival for the 77 patients with SSPT were 74% and 76% respectively. The 18-month and 5-year OS rates of the 77 patients were 72% and 53%, respectively. Table 2 depicts the results of UVA and MVA of 5-year OS including a total of 16 covariates. Cigarette smoking was not specifically analyzed as a risk factor because of the small number of non-smokers (n = 8). Patients with SSPT were further examined in relation to the presence of either esophageal cancer or HCC (due to their poor outcomes when compared with other SSPT subsites; Figs 1 and 2b) (eso-HCC subgroup, n = 8) vs. other tumors (NO eso-HCC subgroup, n = 69). The results of UVA demonstrated that eso-HCC subgroup, pN status, ECS, and lymphatic invasion were significant poor prognostic factors for 5-year OS. After allowance for potential confounders, MVA demonstrated that the eso-HCC subgroup (Fig 2b) and ECS (Fig 2c) retained their independent prognostic significance for 5-year OS (Table 2). Table 3 summarizes the general characteristics of the eight OSCC patients who presented with SSPT located at the esophagus (n = 4) or the liver (n = 4). Of them, five presented with ECS (two cases with esophageal cancer and three with HCC). All of them died either of disease or disease-related causes (i.e., primary OSCC or simultaneous eso-HCC; Table 3, footnote). Of the three cases without ECS, one died of third primary cancer of the tongue base 29 months after treatment of the primary OSCC (case 1). The remaining two patients are still alive after a follow-up of 18 and 36 months, respectively (cases 2 and 5, Table 3)

### Prognostic scoring system for OSCC patients with SSPT

We developed a 3-point prognostic scoring system by summing up the two independent prognostic factors identified in MVA (i.e. eso-HCC subgroup and ECS). A score of 0 was assigned when the risk factor was absent, whereas a score of 1 was given in presence of the risk factor. As expected, high-risk patients with a score of 2 showed the worst prognosis. Moreover, intermediate-risk patients with a score of 1 had worse 5-year OS rates than low-risk patients who scored 0 (Fig 2d).

## Discussion

The choice of the optimal therapeutic modality for OSCC patients who present with SSPT remains problematic. When SSPTs are surgically resectable, it is still unclear whether simultaneous or sequential removal should be pursued [7,8]. In cases treated in a sequential manner, the order by which tumors should be removed (primary OSCC *vs*. SSPT) has not been clearly established. Similarly, there is a lack of consensus on the priority for RT or CCRT in non-surgical cases. Finally, the question as to whether patients with an expected 2-year OS of less than 10% should receive treatment with curative intent or palliation remains open. Starting from these premises, we designed the current study to identify the main prognostic factors in OSCC patients presenting with SSPT and incorporate them into a risk stratification scheme.

In this retrospective study examining the records of 1822 resected OSCC patients enrolled between 1996 and 2014, we identified 77 cases with SSPT treated with curative intent because of the absence of distant metastases at their primary staging. First, our data demonstrate that OSCC patients presenting with SSPT had a lower 5-year OS rate than those without SSPT (53% *vs*. 69%, respectively). Notably, eso-HCC subgroup, pN status, ECS, and lymphatic invasion were identified as significant adverse prognostic factors for 5-year OS. However, only eso-HCC subgroup and neck nodal ECS retained their independent prognostic significance in MVA. According to unpublished data from the Taiwanese National Health Institute (released solely to Taiwan tertiary hospitals and not publicly available), the 3- and 5-year overall survival rates for Taiwanese patients with esophageal cancer and HCC alone are 17%/14% and 39%/28%, respectively (2007–2009). No survival data are currently available for patients with esophageal cancer or HCC according to the presence or absence of co-occurring OSCC. In this study, we identified four patients with co-occurrence of OSCC and esophageal cancer. Only one patient was alive at the date of last follow-up (18 months). Notably, we also identified one patient with co-occurring OSCC and HCC who survived at 36 months. Although the number of cases included was small, it appears that SSPT subsites are critical determinants of survival. According to our prognostic scoring system based on the two independent risk factors, the worst OS rate (0% at 2 years) was observed for patients presenting with both SSPT located at the esophagus or the liver and ECS.

The clinical outcomes of the 77 OSCC patients presenting with SSPT are summarized in Fig 1. After the exclusion of high-risk patients with SSPT located in the esophagus (n = 4) or the liver (n = 4), we found that 79% (61/77) of SSPT were located in the oral cavity, whereas 5% (4/77) originated from the soft palate. All of these patients received simultaneous radical treatment. The remaining four patients with SPT located at the tongue base, thyroid, stomach, and colon were treated with sequential definitive treatment. At the time of the last follow-up, 51% of these patients (n = 35) were alive, whereas the remaining 49% (n = 34) were dead.

One of the main clinical issues for patients in eso-HCC subgroup was that the definite diagnosis of ECS requires ND and subsequent pathological examination. In this scenario, the selection of the optimal treatment strategy (simultaneous *vs*. sequential; definitive *vs*. palliative) poses major challenges. Because of the higher 2-year OS in patients without ECS (66.7% [1/3] *vs*. 0% [5/5], Table 3), we propose a sequential treatment comprising ND to confirm or exclude the presence of neck nodal ECS. In the absence of ECS, definitive treatment with radical surgery should be pursued in eco-HCC subgroup patients. In presence of ECS, the prognosis is dismal and supportive care should be recommended. A reliable imaging or biomarker of ECS is eagerly awaited for OSCC patients with clinically suspected neck nodal metastases and SSPT located at the esophagus or the liver. Interestingly, a previous small-sized FDG-PET study from our group demonstrated that 38 (95%) of the 40 patients with a preoperative maximum standardized uptake value of the neck lymph nodes (SUVnodal-max) ≥5.7 had ECS [15]. Such an imaging biomarker would avoid unnecessary radical neck surgery and promote the use of the best supportive care for patients with poor prognosis.

Some limitations of our study merit comment. First, its retrospective single-center nature limits the generalizability of the results. Although this study is the largest to date in which SSPT has been analyzed in a homogenously treated cohort of OSCC patients enrolled in a single institution, there were no commonly reported lung or hypopharynx SSPT identified in this series. Although primary lung cancer is frequently associated with head and neck malignancies, we believe that there are at least two reasons that may explain its unusually low frequency in our study. First, all of the study participants were scheduled for radical surgery and patients presenting with lung lesions (either primary or metastatic) were excluded. Second, we identified 29 patients as having a second primary lung cancer after at least one month from the initial OSCC diagnosis. However, they were included in the Not-SSPT subgroup and not among patients presenting SSPT (Fig 1) based on the definition used for the current study (i.e., SSPT defined as two independent cancers identified within one month of each other). Another caveat is that we did not collect the occurrence of malnourishment, a major factor influencing OS. In addition, our OSCC patients without ECS had earlier-stage esophageal cancer when compared with those showing ECS (Table 3). Finally, all of the participants came from an area in which betel quid chewing is endemic; therefore, the findings might not be applied to patients in different geographic locations.

**Fig 3:**
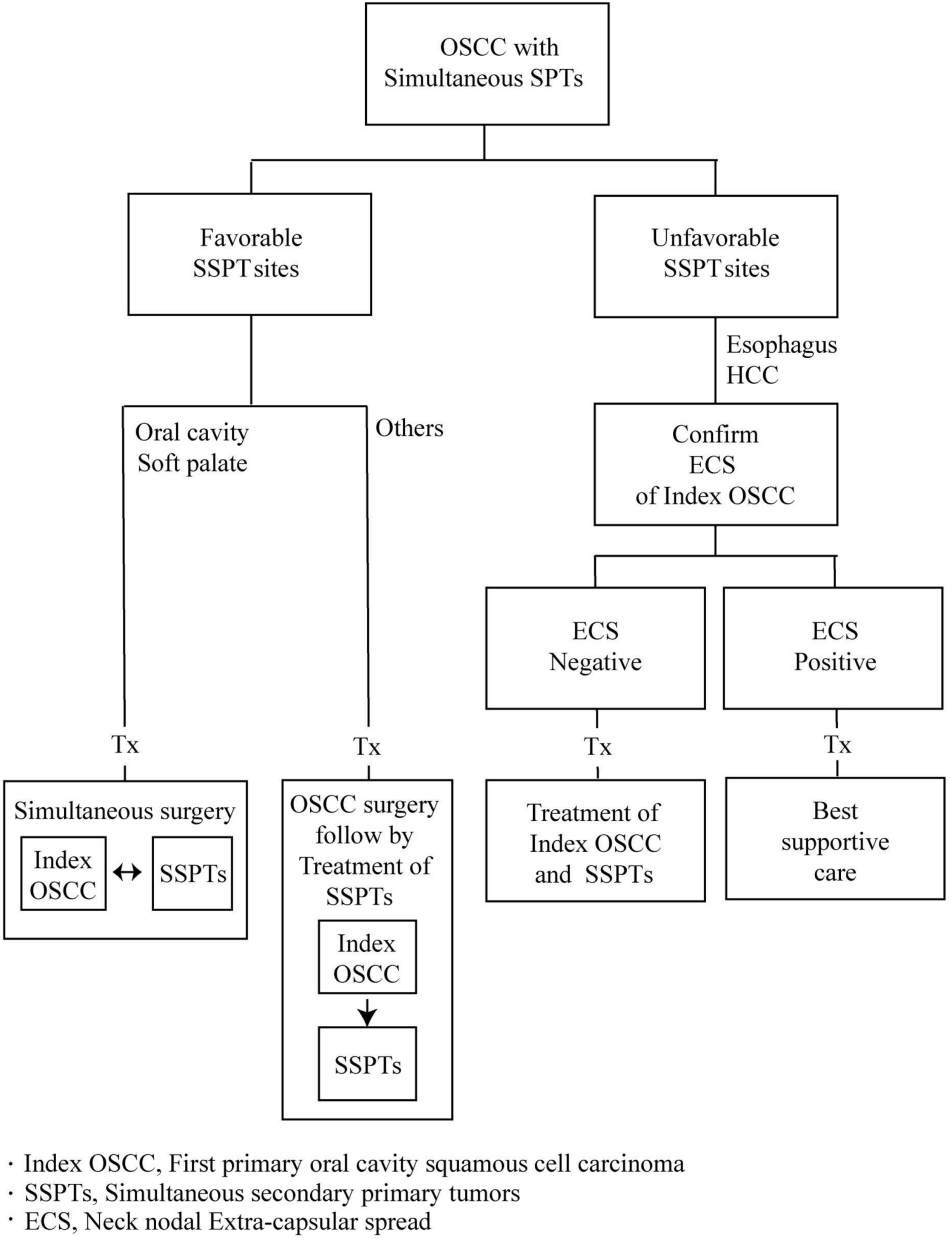
Flowchart of treatment selection in OSCC patients presenting with SSPT.

In summary, the results of our study indicate that radical surgery (either with simultaneous or sequential definitive treatment) should be recommended for OSCC patients who present with SSPT but who do not carry adverse risk factors (neck nodal ECS or eso-HCC subgroup). In the eso-HCC subgroup, the presence or absence of ECS should be investigated by means of ND or other reliable methods. In the absence of ECS, sequential definitive treatment should be recommended. Because the presence of ECS portends a poor prognosis, the use of best supportive care (instead of sequential definitive treatment) is indicated to improve the quality of life unless other novel treatments are discovered in the next future (Fig 3).

## Supporting Information

S1 Data Dataset. (XLS)

S2 Data Dataset specification. (XLS)
